# STEADI in Primary Care: A Process Evaluation of the New York State Implementation

**DOI:** 10.1093/geroni/igaa057.2800

**Published:** 2020-12-16

**Authors:** Yvonne Johnston, Chelsea Reome-Nedlik

**Affiliations:** 1 Binghamton University, Binghamton, New York, United States; 2 Broome County Health Department, Binghamton, New York, United States

## Abstract

This session presents findings from a STEADI process evaluation that was conducted within a primary care setting in New York State. This process evaluation used mixed methods including quantitative analysis of surveys with clinic staff as well as qualitative methods such as intercept interviews with healthcare providers and clinic staff, and structured interviews with key stakeholders. The RE-AIM framework guided development of the process evaluation tools. The process evaluation was conducted over a 2-month period approximately 18 months post-implementation. Facilitators included: (a) Adoption - physician champion and administrative support; (b) Implementation - wellness coordinators, preparation and training, and organizational quality measures; (c) Maintenance - feedback from patients, local and national recognition, and impact on fall-related outcomes. Barriers included: (a) Adoption – organizational priorities and complexity of electronic health records; (b) Implementation – resistance to change and competing patient care demands; (c) Maintenance - staff turnover and follow through of referrals.

